# Peak Flow Meter and Spacer Use in Adolescents with Asthma: More than Just Ownership

**DOI:** 10.3390/children13020302

**Published:** 2026-02-22

**Authors:** Hyekyun Rhee, Nattasit Katchamat

**Affiliations:** 1 School of Nursing, University of Texas at Austin, 1710 Red River St., Austin, TX 78712, USA; nk24573@my.utexas.edu; 2 Ramathibodi School of Nursing, Faculty of Medicine Ramathibodi Hospital, Mahidol University, 270, Rama 6, Rachathewi, Bangkok 10400, Thailand

**Keywords:** adolescent, asthma, peak flow meter, inhalation spacers, exacerbation, asthma control

## Abstract

Background: Effective self-management is essential for optimizing asthma control. Although peak flow meters (PFMs) and spacers are recommended to support self-management, the associations between device use patterns and asthma outcomes remain unclear. This study aims to examine the ownership and use of devices among adolescents with asthma and their associations with asthma exacerbations in the past 12 months and asthma control. Methods: Cross-sectional data from 373 inner-city adolescents with asthma were analyzed. Participants reported PFM or spacer ownership and their frequency of use. Exacerbation history was determined based on oral corticosteroid use, hospitalizations, and emergency department visits in the past 12 months. Asthma control was assessed using the Asthma Control Questionnaire. Chi-square tests and independent *t*-tests were used to examine associations among device ownership, use frequency, asthma control, and exacerbations. Results: Ownership rates were 36% for PFMs and 61.6% for spacers. Ownership of both devices was negatively associated with asthma exacerbations in the past year (all *p* < 0.001). Regular use was reported by only 15.2% of PFM owners and 40.2% of spacer owners. Regular users did not differ from infrequent users in exacerbation history but reported significantly poorer asthma control (PFM: *p* = 0.007; spacer: *p* < 0.001). Conclusions: Adolescents’ ownership of peak flow meters and spacers remains suboptimal, and the routine adoption of these devices is limited. Adolescents with exacerbations in the past year were more likely to own devices but use them infrequently. Regular use was associated with poorer asthma control, suggesting reactive rather than preventive use. Findings highlight the need for improved education on preventive device use, enhanced training in proper use, and proactive integration of devices into adolescent asthma self-management.

## 1. Introduction

Asthma is a leading chronic condition in adolescents, affecting nearly two million U.S. youths aged 12–17 [1]. Effective self-management is essential for achieving optimum asthma control and reducing asthma-related burden on adolescents and society. To support asthma self-management, the use of peak flow meters (PFMs) and spacers has been recommended by both the Global Initiative for Asthma (GINA) [2] and the National Asthma Education and Prevention Program (NAEPP) [3]. PFMs are particularly encouraged for individuals with a history of exacerbation, moderate-to-severe asthma, or poor perception of airflow obstruction, as they help detect early signs of worsening asthma and evaluate the response to treatment changes [3]. Spacers are used to optimize medication delivery from metered-dose inhalers to the lungs and reduce the risk of local side effects, such as dysphonia and oral candidiasis [2], particularly in children [4].

Studies have reported positive effects of peak flow monitoring on asthma outcomes in pediatric patients [5,6,7,8]. Similarly, spacer use has been associated with improved asthma control [9,10], better lung function [10,11], and reduced acute healthcare utilization among children with asthma [12]. However, some studies have challenged the clinical effectiveness of these tools in improving asthma control [13,14,15,16,17]. The inconsistent findings may be partly due to the failure to consider the actual usage patterns of these devices. Use of PFMs or spacers is infrequent and often poorly integrated into daily asthma management routines [5,18].

Few studies have clearly distinguished between ownership and usage patterns of these devices when examining their relationships with asthma outcomes. To date, the direct relationship between regular device use and asthma control remains unclear. Moreover, the available literature has largely assessed device use within clinical trial settings, where education and supervision are provided. Less is known about real-world patterns of PFM and spacer use, particularly in community-based, underserved adolescents. To address these limitations in the literature, the current study aimed to assess the ownership and use of PFMs and spacers among urban, predominantly minoritized adolescents with asthma and to examine their associations with exacerbation and asthma control. We hypothesized that device ownership and usage patterns would be associated with asthma control and asthma exacerbations.

## 2. Methods

This study presents a cross-sectional analysis of baseline data from a randomized controlled trial evaluating the effectiveness of a peer-led asthma self-management program (ClinicalTrials.gov Identifier: NCT02293499). The trial was conducted in three major urban U.S. cities between 2016 and 2020.

### 2.1. Study Sample

This study included 373 adolescents aged 12–20 years with persistent asthma recruited through clinician or school referrals, word-of-mouth, and direct recruitment at community venues or clinics. Eligibility included physician-diagnosed asthma for at least one year, asthma-related healthcare utilization within the 12 months preceding enrollment, and persistent asthma according to NAEPP guidelines [3]. Only those who could read and speak English were included in the study. Adolescents with other chronic comorbid conditions, as reported by parents, were excluded to avoid confounding factors that could complicate asthma self-management routines.

### 2.2. Data Collection

Peak Flow Meter and Spacer Use: A survey was administered to assess ownership and usage patterns of both peak flow meters and spacers. Participants were asked whether they owned each device with the question, “Do you have a peak flow meter [or spacer]?” using a binary response option (“Yes” or “No”). For those who reported owning a device, frequency of use was assessed with four response options: *Daily*, *Several times a week*, *Occasionally*, and *Rarely*. For analysis, responses were dichotomized: *Daily* and *Several times a week* were categorized as “Regular use”, while *Occasionally* and *Rarely* were categorized as “Infrequent use.”

Asthma Exacerbation: Asthma exacerbations were defined as the occurrence of any one of three acute asthma-related events in the past 12 months [19]: (1) use of oral corticosteroids (OCSs) for at least three consecutive days, (2) hospitalization for asthma, and (3) emergency department (ED) visits. A binary exacerbation variable was created to indicate whether any of these events had occurred. Each event was also examined individually in relation to device ownership and use.

Asthma Control: Asthma control was measured using the 6-item Asthma Control Questionnaire (ACQ) [20], which assesses symptom control during the past week on a 7-point scale ranging from 0 (no symptoms) to 6 (very severe symptoms). The items evaluate nighttime symptoms, daytime symptoms, activity limitation, and the frequency of rescue medication use. A mean score was calculated, with higher scores indicating poorer asthma control. Internal consistency reliability for the ACQ in this sample was high (Cronbach’s α = 0.86).

Asthma Severity: Asthma severity was assessed using a 4-time survey reflecting impairment criteria including symptoms, nighttime awakenings, activity limitations and short-acting beta-agonist (SABA) use in the past 4 weeks. Each item was rated on a 4-point scale capturing increasing frequency and intensity, aligned with national asthma guidelines’ classifications for severity [3]. Based on these responses, severity was categorized as mild intermittent, mild persistent, moderate persistent, or severity persistent. Asthma severity was dichotomized into two groups: mild asthma (combining the two lower severity levels) and moderate-to-severe asthma (combining the two higher severity levels).

### 2.3. Ethical Considerations

The study protocol was reviewed and approved by the Institutional Review Boards of the participating academic institutions. Written informed consent was obtained from parents or from adolescents aged 18 and older, and assent was obtained from younger participants prior to data collection.

### 2.4. Data Analysis

Data were analyzed using SPSS (v.29.0). Frequencies and percentages were computed for categorical variables including device ownership and dichotomized usage pattern. Pearson chi-square (χ^2^) tests were conducted to examine associations between device ownership and usage patterns (peak flow meter and spacer) and events of asthma exacerbation (OCS use, hospitalization, and ED visits), as well as healthcare insurance type. Fisher’s exact test was used to examine the association between categorical variables if more than 20% of cells had expected frequencies less than 5 [21]. Independent samples *t*-tests were used to compare mean differences in asthma control and age between device owners and non-owners and between regular and infrequent device users. The effect size (Cohen’s d and Phi (ϕ)) was computed to assess the magnitude of associations. Linear regressions were conducted to examine the association between device ownership and usage patterns (regular vs. infrequent use) and asthma control, whereas logistic regressions were used to evaluate their associations with exacerbation outcomes and asthma severity. Significance was set at two-sided *p* < 0.05 for all tests.

## 3. Results

Males and females were equally distributed in the sample. The majority were Black or African American (78.6%), and most participants (71%) were covered by public health insurance. The mean age of the sample was 14.68 years (SD = 1.93). Table 1 provides the demographic characteristics of the sample. Further detailed sample characteristics are reported elsewhere [22].

**Peak Flow Meter (PFM) Ownership and Usage Patterns**:

Overall, 36% (n = 134) of participants reported owning a PFM. Those who owned peak flow meters were significantly younger than non-owners (t = 2.71; *p* = 0.007), while no associations were found between ownership and sex or type of healthcare insurance (Table 1). Ownership was significantly associated with a history of asthma exacerbations. Significantly higher PFM ownership was reported by participants who had used OCSs (χ^2^ = 20.12; *p* < 0.001); been hospitalized (χ^2^ = 13.88; *p* < 0.001); visited the ED (χ^2^ = 18.16; *p* < 0.001) in the past 12 months; or moderate-to-severe persistent asthma (χ^2^ = 4.58; *p* < 0.032) compared to each of their counterparts. Overall, those who had experienced exacerbation reported higher PFM ownership than their non-exacerbation counterparts (χ^2^ = 16.76; *p* < 0.001). After controlling for age, PFM owners were more likely to have past-year OCS use (OR: 2.83; 95% CI: 1.76–4.53; *p* < 0.001), past-year hospitalization (OR: 2.77; 95% CI: 1.53–5.01; *p* < 0.001), past-year ED visits (OR: 2.65; 95% CI: 1.67–4.23; *p* < 0.001), and experienced an exacerbation (OR: 2.36; 95% CI: 1.52–3.65; *p* < 0.001). However, asthma control was not significantly associated with PFM ownership status (t = −0.338; *p* = 0.74) (Table 1). Likewise, after controlling for age, asthma severity did not significantly predict PFM ownership (OR: 1.48; 95% CI: 0.96–2.28; *p* < 0.079).

Among PFM owners, only 15% (n = 20/132) reported using it daily or several times a week. (Note, two of the PFM owners did not provide usage data.) Usage patterns did not differ by participant age, sex or history of asthma exacerbations (Table 2). However, regular users were significantly more likely to have moderate-to-severe persistent asthma (χ^2^ = 6.69; *p* = 0.01) and poorer asthma control (*t* = −2.76; *p* = 0.007) than infrequent users (Table 2). Logistic and linear regression analyses revealed that those with moderate-to-severe persistent asthma were 4.2 times more likely to report regular use of PFMs than those with mild severity (OR: 4.22; 95%CI: 1.33–13.43; *p* = 0.015). Similarly, regular users reported significantly poorer asthma control (B = 0.81; SE = 0.29; *p* = 0.007) than infrequent users.

**Spacer Ownership and Usage Patterns**:

Nearly all participants (98%) reported using a short-acting beta-agonist (SABA) delivered via metered-dose inhaler in the past 2 weeks. Nonetheless, spacer ownership was reported by only 60.8% (n = 227) of participants. While those who owned spacers were younger than non-owners (t = 4.83; *p* < 0.001), ownership was not associated with sex or healthcare insurance type (Table 1). Like PFMs, spacer ownership was higher among those with past-year exacerbation than among those without exacerbation (χ^2^ =10.84; *p* < 0.001). Specifically, ownership was higher among those who had used OCSs (χ^2^ = 9.04; *p* = 0.003); been hospitalized (χ^2^ = 8.13; *p* = 0.004); or had an ED visit (χ^2^ = 14.49; *p* < 0.001). Compared to non-owners, spacer owners were more likely to have past-year OCS use (OR: 2.09; 95% CI: 1.26–3.48; *p* = 0.004), past hospitalization (OR: 2.36; 95% CI: 1.18–4.71; *p* = 0.015), past-year ED visits (OR: 2.53; 95% CI: 1.51–4.23; *p* < 0.001) and at least one of the exacerbation events (OR: 1.95; 95% CI: 1.25–3.06; *p* = 0.004) after adjusting for age. However, current asthma control was not significantly different between those who owned spacers and non-owners (t = −0.031; *p* = 0.976) (Table 1). Likewise, spacer ownership did not differ by asthma severity (χ^2^ = 2.99; *p* = 0.084).

Among spacer owners, only 40% (n = 90/224) were regular users, using the spacer daily or several times a week (Table 2). Regular users were younger than infrequent users (*t* = 3.25; *p* < 0.001). No significant sex difference was observed in usage frequency. Compared to infrequent users, regular spacer users were more likely to have used OCSs in the past 12 months (χ^2^ = 4.62; *p* = 0.032) and to have moderate-to-severe persistent asthma (χ^2^ = 10.95; *p* < 0.001), whereas they did not differ in hospitalization or ED visit rates. Similarly to PFMs, regular spacer users reported significantly poorer asthma control than infrequent users (*t* = −4.46; *p* < 0.001) (Table 2). After controlling for age and insurance, regular spacer users were more likely to have past-year OCS use (OR: 1.80; 95% CI: 1.00–3.24; *p* = 0.049) and moderate-to-severe persistent asthma (OR: 2.11; 95% CI: 1.18–3.75; *p* = 0.011) than infrequent users. Moreover, after controlling for age and insurance, regular users also reported higher asthma control scores (i.e., poorer asthma control) than infrequent users (B = 0.63; SE = 0.16; *p* < 0.001).

### Effect Size Analysis

**Figure 1:**
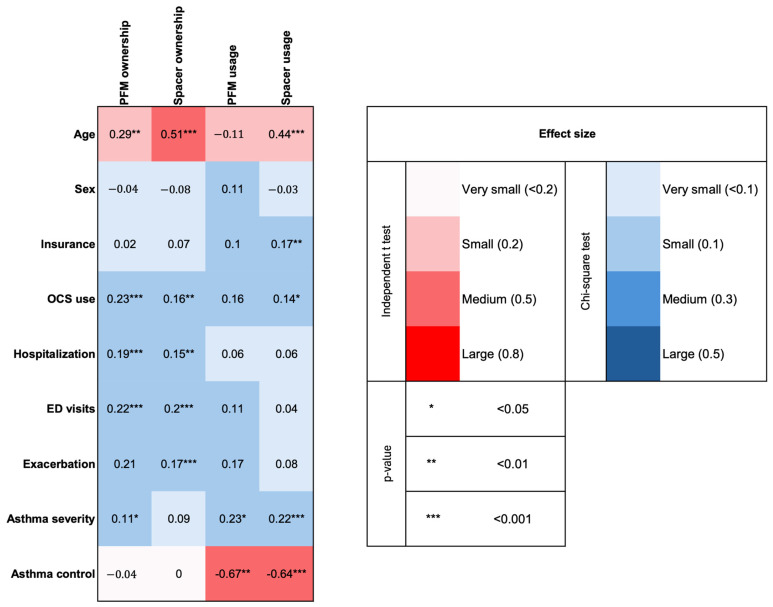
Heatmap illustrating the effect sizes of the associations between the ownership and usage patterns of peak flow meters and spacers, and demographics and asthma outcomes among adolescents. Values are based on independent samples *t*-tests (red color) and chi-square tests (blue color). PFM = peak flow meter, OCS = oral corticosteroid, and ED = emergency department.

The heatmap in Figure 1 illustrates the effect sizes and statistical significance of the associations between device ownership and usage patterns, demographic characteristics, and asthma outcomes. The heatmap shows small and medium effect sizes for the associations between device ownership and age (for PFMs *d* = 0.29; for spacers *d* = 0.51), while small effects were noted for the associations between usage patterns and age (for PFMs *d* = −0.11; for spacers *d* = 0.44). The heatmap also reveals moderate effect sizes for associations between device usage patterns and asthma control (for PFMs *d* = −0.67; for spacers *d* = −0.64). Effect sizes for the associations between device ownership and exacerbation-related variables were small (ϕ = 0.15 to 0.23), whereas those between usage patterns and exacerbation-related variables were mostly very small (ϕ = 0.04 to 0.17). Small effect sizes were also observed for the association between device ownership and usage patterns, and asthma severity (ϕ = 0.11 to 0.23), except for spacer ownership and asthma severity, which showed a very small effect size (ϕ = 0.09).

## 4. Discussion

We examined the ownership and usage patterns of peak flow meters (PFMs) and spacers among inner-city adolescents with asthma. Overall, slightly over one-third of participants reported owning a PFM, while nearly two-thirds owned a spacer. Owners of either of these devices were younger than those who did not own one. In examining usage patterns, we found no age-related differences in the frequency of PFM use, which contrasts with a previous study reporting younger age as a predictor of regular peak flow meter use in children [23]. For spacers, however, regular users were younger than infrequent users. Our findings regarding spacer ownership and usage, both of which declined with increasing age, are consistent with prior research showing higher rates of spacer ownership and use among younger children compared with older ones [24]. The lower ownership and less frequent use of spacers among older children may reflect increasing confidence in using inhalers independently [24] as they assume greater autonomy in asthma management. In addition, younger adolescents are often more closely supervised by parents [24], who may encourage or facilitate spacer use. The lack of age differences in PFM use suggests that monitoring behaviors may decline uniformly across adolescence or that PFMs are less integrated into routine asthma care regardless of age. Given the potential benefits of these devices in asthma management, targeted interventions are needed to enhance awareness and promote routine use among older adolescents, who may be at greater risk for underutilization.

Our data suggest that adolescents who experienced exacerbations in the past 12 months are twice as likely to receive these devices compared to those with no history of exacerbation, likely as part of post-acute care management. This pattern reflects a reactive approach in clinical practice, where devices are prescribed after exacerbations rather than as part of proactive asthma self-management strategies. It may also suggest that patients or families delayed purchasing these devices until perceived need increased following an exacerbation, highlighting the crucial role of patient or family perceptions in device adoption. Notably, only 48% of adolescents with exacerbation owned a PFM, despite the NAEPP’s recommendation to use PFMs for asthma monitoring in individuals with a history of exacerbation [3].

We learned that the ownership of these devices did not always translate to their regular use. Merely 15% of PFM owners and 40% of spacer owners reported using the devices daily or several times a week. These low rates of regular use are consistent with prior studies reporting children’s limited adherence to PFMs [5,25]. Notably, regular users of both PFMs and spacers did not differ from infrequent users in past-year exacerbation history, except for the fact that regular spacer users were more likely to have used OSCs in the past year than infrequent users.

We also found no significant differences in ownership of PFMs or spacers by asthma severity; however, participants with moderate-to-severe persistent asthma were more likely to use the devices regularly compared to those with mild severity. This pattern suggests that while clinicians may prescribe the devices similarly across severity levels, adolescents with moderate-to-severe persistent asthma may perceive greater vulnerability, which in turn motivates more consistent use.

Simply owning these devices was not associated with better asthma control, whereas regular users reported significantly poorer asthma control. This aligns with McMullen et al. [23], who found that greater symptom frequency predicted consistent peak flow meter use. These findings suggest that adolescents experiencing greater symptom burden tend to use these devices more regularly to manage uncontrolled symptoms rather than as a preventive strategy. In other words, consistent device use may reflect underlying asthma severity rather than indicating effective self-management. In contrast, several studies have demonstrated that incorporating peak flow monitoring into asthma management can lead to improvements in asthma outcomes and medication adherence over time [26,27,28,29]. The seemingly inconsistent findings between the current study and the earlier studies likely reflect differences in study designs—retrospective assessment of asthma outcomes in the present study versus prospective evaluation in prior research. These differences further underscore the importance of proactive peak flow meter use, particularly for youth with high symptom levels, as a means of preventing worsening asthma. To maximize the benefits of these devices, however, it is critical to provide proper education, skill training and ongoing reinforcement to support consistent and effective use.

Overall, device ownership was not associated with the type of healthcare insurance, suggesting comparable coverage for these devices under both private and public plans. Interestingly, both spacer ownership and regular use were significantly associated with younger age. These findings suggest that providers may be more likely to prescribe spacers to younger adolescents, for whom parents are more likely to fill the prescriptions and supervise and reinforce its use, which, in turn, may contribute to greater adherence to spacer use among younger teens. As adolescents become older and gain greater independence, it may become more difficult to reinforce consistent spacer use, unless its use is routinized as part of their self-management behavior during earlier years.

### 4.1. Strengths and Limitations

To our knowledge, this study is the first study to systematically examine the relationships among peak flow meter and spacer ownership, usage patterns, patient characteristics, and asthma control in adolescents. The study offers important insights into the extent to which these devices are distributed and used among urban, primarily minoritized adolescents with asthma. In addition, we identified several demographic and asthma morbidity factors associated with both the ownership and usage patterns of these devices, for which the existing literature is extremely limited.

This study has several limitations that warrant caution in interpretation. First, the cross-sectional design precludes causal inference between device ownership and use and asthma morbidity factors. Second, the exclusive reliance on self-reported data introduces the potential for recall bias, reporting bias or social desirability bias, particularly regarding device use frequency and past-year exacerbations. Third, we did not assess device technique, which may partly explain the absence of or negative associations between device use and asthma control. Fourth, this study did not collect data on whether these devices were prescribed by healthcare providers, which may be important for understanding the suboptimal ownership observed in this sample. Also, this study did not assess psychological and behavioral factors that may influence adolescents’ adherence to these devices, nor did it capture the specific roles of individual healthcare team members in optimal device adoption and adherence. Additionally, the generalizability of our findings may be limited by the convenience sample of predominantly urban, Black or African American adolescents. Finally, because the data were collected more than five years ago, they may be viewed as dated. Despite these limitations, our study provides valuable insights into the adoption of peak flow meters and spacers among urban minority adolescents with asthma—a topic that has received little focused attention in the literature.

### 4.2. Implications for Future Research and Practice

Future research should employ longitudinal designs to clarify the temporal relationships between the use of peak flow meters and/or spacers and asthma outcomes in adolescents. Such designs would allow examination of the extent to which regular use of these devices, as part of asthma self-management, contributes to improved asthma control over time. In addition, replicating our findings in different geographic regions or in larger, more diverse adolescent populations would further strengthen generalizability. The development and evaluation of interventions aimed at promoting appropriate and consistent use of PFMs and spacers, through education, skill training, and behavioral reinforcement, may play a critical role in improving responsible asthma management and reduce asthma morbidity among adolescents.

Relatively low ownership of these devices underscores the need for more rigorous efforts to increase equitable access among urban minority adolescents, who experience greater asthma-related morbidity than their white counterparts. In addition, adolescents’ suboptimal routine use highlights the importance of periodic monitoring and implementation of strategies that reinforce integrating these devices into regular asthma self-management.

Moreover, prospective data collection, combined with objective, technology-assisted methods for monitoring and tracking device use and asthma outcomes, such as smartphone apps designed to support device usage, digital peak flow meters, and smart spacers, could reduce recall bias and enhance measurement precision. Indeed, emerging digital health approaches highlight important directions for advancing adolescent asthma self-management beyond traditional, user-dependent monitoring strategies such as peak flow meters. For example, the ALEX project developed by the Basel Research Centre for Child Health illustrates how smartphone-based digital health assistants can integrate passive biomarker monitoring and meaningful youth engagement to reduce user burden while promoting sustained engagement [30]. In parallel, international frameworks such as ARIA 2024 emphasize the growing role of digitally enabled, artificial intelligence-assisted care pathways in adherence monitoring support, personalized feedback, and timely clinical intervention across airway diseases [31]. Evidence from other chronic respiratory conditions, including allergic rhinitis, further demonstrates the feasibility of using digital tools to capture real-world adherence patterns and inform treatment strategies at scale [32]. Given a persistent gap between methods for asthma monitoring and medication delivery, and supportive device ownership and usage, this study underscores the need for forward-looking strategies involving developmentally engaging and low-burden digital technologies to enhance adherence to asthma self-management among adolescents.

Clinicians are uniquely positioned to implement and support these efforts. Clinicians should be more diligent not only in prescribing these devices to adolescents but also in confirming that prescriptions are filled. In addition, providers should identify and address barriers to prescription fulfillment to ensure the distribution of these devices for all adolescents. During clinical encounters, they can assess adolescents’ device use, evaluate device technique and adherence, and provide tailored guidance to motivate and ensure continuous and correct use, thereby maximizing the benefits of these devices in asthma management. PFMs and spacers are particularly valuable following changes in medication regimens as spacers facilitate optimal delivery of new medications to the lungs, while peak flow meters help clinicians monitor the medication effects on lung function by tracking peak flow patterns.

## 5. Conclusions

Adolescents’ access to peak flow meters and spacers remains suboptimal, and the routine adoption of these devices as part of daily asthma management is limited. Also, devices appear to often be provided reactively in response to exacerbations, which represents missed opportunities for proactive asthma care. Moreover, once obtained, they are often used inconsistently, and even regular users may not experience the intended benefits, as reflected by poorer asthma control. Collectively, these findings underscore the need for proactive, multifaceted interventions that extend beyond device distribution and focus on improving adolescents’ understanding, motivation, and integration of these tools into their daily self-management routines to unlock the long-term benefits.

## Figures and Tables

**Table 1:** The peak flow meter and spacer ownerships and asthma outcomes.

Variables		Peak Flow Meter (n = 373)				Spacer (n = 373)			
		Yes, n (%)	No, n (%)	*p*	Effect Size	Yes, n (%)	No, n (%)	*p*	Effect Size
Device ownership		134 (36%)	239 (64%)	-	-	227 (60.8%)	146 (39.2%)	-	-
Age, M (SD)		14.32 (1.95)	14.88 (1.90)	0.007	0.29	14.30 (1.83)	15.27 (0.95)	<0.001	0.51
Female		63 (16.89%)	123 (32.98%)	0.410	−0.04	106 (28.42%)	80 (21.45%)	0.127	−0.08
Insurance	Public insurance	97 (27.02%)	163 (45.40%)	0.731	0.02	167 (46.52%)	93 (25.91%)	0.181	0.07
	Private insurance	35 (9.75%)	64 (17.83%)			56 (15.60%)	43 (11.98%)		
Past-year OCS ^1^ use		56 (15.01%)	48 (12.87%)	<0.001	0.23	76 (20.38%)	28 (7.51%)	0.003	0.16
Past-year hospitalization		32 (8.58%)	23 (6.17%)	<0.001	0.19	43 (11.53%)	12 (3.22%)	0.004	0.15
Past-year ED ^2^ visits		57 (15.28%)	51 (13.67%)	<0.001	0.22	82 (21.98%)	26 (6.97%)	<0.001	0.20
Exacerbation	Yes	74 (19.84%)	80 (21.45%)	<0.001	0.21	109 (29.22%)	45 (12.06%)	<0.001	0.17
	No	60 (16.09%)	159 (42.63%)			118 (31.64%)	101 (27.08%)		
Asthma severity	Mild	62 (16.62%)	139 (37.27%)	0.032	0.11	114 (30.56%)	87 (23.32%)	0.084	0.09
	Moderate-to-severe	71 (19.03%)	100 (26.81%)			112 (30.03%)	59 (15.82%)		
Asthma control, M (SD)		1.46 (1.24)	1.42 (1.05)	0.736	−0.04	1.44 (1.20)	1.43 (1.00)	0.976	−0.00

^1^ OCS = oral corticosteroid. ^2^ ED = emergency department.

**Table 2:** The peak flow meter (PFM) and spacer usage patterns and asthma outcomes among adolescents.

Variables		Peak Flow Meter (n = 132)				Spacer (n = 224)			
		Regular User, n (%)	Infrequent User, n (%)	*p*	Effect Size	Regular User, n (%)	Infrequent User, n (%)	*p*	Effect Size
Device usage		20 (15%)	112 (85%)	-	-	90 (40%)	134 (60%)	-	-
Age, M (SD)		14.50 (1.43)	14.28 (2.04)	0.554	−0.11	13.81 (1.71)	14.60 (1.85)	<0.001	0.44
Female		12 (9.09%)	50 (37.88%)	0.205	0.11	40 (17.86%)	64 (28.57%)	0.626	−0.03
Insurance	Public insurance	16 (12.31%)	80 (61.54%)	0.398 ^†^	0.10	75 (33.94%)	91 (41.18%)	0.01	0.17
	Private insurance	3 (2.31%)	31 (23.85%)			14 (6.33%)	41 (18.55%)		
Past-year OCS ^1^ use		12 (9.09%)	43 (32.58%)	0.071	0.16	38 (16.96%)	38 (16.96%)	0.032	0.14
Past-year hospitalization		6 (4.55%)	26 (19.70%)	0.514	0.06	20 (8.93%)	23 (10.27%)	0.346	0.06
Past-year ED ^2^ visits		11 (8.33%)	45 (34.09%)	0.217	0.11	35 (15.63%)	47 (20.98%)	0.561	0.04
Exacerbation	Yes	15 (11.36%)	58 (43.94%)	0.054	0.17	48 (21.43%)	61 (27.23%)	0.252	0.08
	No	5 (3.79%)	54 (40.91%)			42 (18.75%)	73 (32.59%)		
Asthma severity	Mild	4 (3.03%)	57 (43.18%)	0.01	0.23	33 (14.73%)	80 (35.71%)	<0.001	0.22
	Moderate-to-severe	16 (12.12%)	54 (40.91%)			56 (25.00%)	54 (24.11%)		
Asthma control, M (SD)		2.13 (1.38)	1.32 (1.78)	0.007	−0.67	1.87 (1.33)	1.14 (1.01)	<0.001	−0.64

^1^ OCS = oral corticosteroid. ^2^ ED = emergency department. ^†^ One cell (25%) had expected frequencies less than 5; therefore, Fisher’s exact test was used. The test value was not provided because the test is based on the hypergeometric distribution rather than an approximation, such as the chi-square test.

## Data Availability

The datasets used and analyzed in this paper are available from the corresponding author on reasonable requested. The data are not publicly available due to privacy and ethical restriction.

## References

[1] Center for Disease Control and Prevention Most Recent Asthma Data. https://www.cdc.gov/asthma-data/about/most-recent-asthma-data.html.

[2] Global Initiative for Asthma Global Strategiy for Ashtma Management and Prevention. http://www.ginasthma.org.

[3] National Heart Lung and Blood Institute (2007). Expert Panel Report 3: Guidelines for the Diagnosis and Management of Asthma.

[4] Newman S.P. (2004). Spacer Devices for Metered Dose Inhalers. Clin. Pharmacokinet..

[5] Yoos H.L., Kitzman H., McMullen A., Henderson C., Sidora K. (2002). Symptom monitoring in childhood asthma: A randomized clinical trial comparing peak expiratory flow rate with symptom monitoring. Ann. Allergy Asthma Immunol. Off. Publ. Am. Coll. Allergy Asthma Immunol..

[6] Tinkelman D., Schwartz A. (2004). School-based asthma disease management. J. Asthma.

[7] Burkhart P.V., Rayens M.K., Revelette W.R., Ohlmann A. (2007). Improved health outcomes with peak flow monitoring for children with asthma. J. Asthma.

[8] Bheekie A., Syce J.A., Weinberg E.G. (2001). Peak expiratory flow rate and symptom self-monitoring of asthma initiated from community pharmacies. J. Clin. Pharm. Ther..

[9] Levy M.L., Hardwell A., McKnight E., Holmes J. (2013). Asthma patients’ inability to use a pressurised metered-dose inhaler (pMDI) correctly correlates with poor asthma control as defined by the global initiative for asthma (GINA) strategy: A retrospective analysis. Prim. Care Respir. J..

[10] Schor D., Rizzo J.A., Medeiros D., Dela Bianca A.C., Silva A.R., Nunes C., Morais-Almeida M., Sarinho E. (2017). Home-made spacer as an auxiliary device in administration of beclomethasone via pressurized metered dose inhaler for asthma control. A randomized controlled pragmatic trial. Respir. Med..

[11] Dahiya B., Mathew J.L., Singh M. (2007). Randomized trial of spacers in asthma. Indian J. Pediatr..

[12] Yildirim M., Griffin P., Keskinocak P., O’Connor J.C., Swann J.L. (2021). Estimating the impact of self-management education, influenza vaccines, nebulizers, and spacers on health utilization and expenditures for Medicaid-enrolled children with asthma. J. Asthma.

[13] Brouwer A.F.J., Roorda R.J., Brand P.L.P. (2006). Home spirometry and asthma severity in children. Eur. Respir. J..

[14] Slieker M.G., van der Ent C.K. (2003). The diagnostic and screening capacities of peak expiratory flow measurements in the assessment of airway obstruction and bronchodilator response in children with asthma. Monaldi Arch. Chest Dis..

[15] Ammari W.G., Toor S., Chetcuti P., Stephenson J., Chrystyn H. (2015). Evaluation of asthma control, parents’ quality of life and preference between AeroChamber Plus and AeroChamber Plus Flow-Vu spacers in young children with asthma. J. Asthma.

[16] Burgess S.W., Sly P.D., Cooper D.M., Devadason S.G. (2007). Novel spacer device does not improve adherence in childhood asthma. Pediatr. Pulmonol..

[17] Guilbert T.W., Colice G., Grigg J., van Aalderen W., Martin R.J., Israel E., Postma D.S., Roche N., Phipatanakul W., Hillyer E.V. (2017). Real-Life Outcomes for Patients with Asthma Prescribed Spacers for Use with Either Extrafine- or Fine-Particle Inhaled Corticosteroids. J. Allergy Clin. Immunol. Pr..

[18] van der Meer V., Rikkers-Mutsaerts E.R., Sterk P.J., Thiadens H.A., Assendelft W.J., Sont J.K. (2006). Compliance and reliability of electronic PEF monitoring in adolescents with asthma. Thorax.

[19] Fuhlbrigge A., Peden D., Apter A.J., Boushey H.A., Camargo C.A., Gern J., Heymann P.W., Martinez F.D., Mauger D., Teague W.G. (2012). Asthma outcomes: Exacerbations. J. Allergy Clin. Immunol..

[20] Juniper E.F., O’Byrne P.M., Guyatt G.H., Ferrie P.J., King D.R. (1999). Development and validation of a questionnaire to measure asthma control. Eur. Respir. J..

[21] Kim H.Y. (2017). Statistical notes for clinical researchers: Chi-squared test and Fisher’s exact test. Restor. Dent. Endod..

[22] Rhee H., Love T., Harrington D., Walters L. (2020). Comparing Three Measures of Self-Efficacy of Asthma Self-Management in Adolescents. Acad. Pediatr..

[23] McMullen A.H., Yoos H.L., Kitzman H. (2002). Peak flow meters in childhood asthma: Patient report of use and perceived usefulness. J. Pediatr. Health Care.

[24] Volerman A., Fierstein J., Boon K., Vojta D., Gupta R. (2021). Determinants of asthma knowledge and practices among caregivers of children with moderate-to-severe persistent asthma. Ann. Allergy Asthma Immunol..

[25] Burkhart P.V., Dunbar-Jacob J.M., Fireman P., Rohay J. (2002). Children’s adherence to recommended asthma self-management. Pediatr. Nurs..

[26] Farzandipour M., Heidarzadeh Arani M., Sharif R., Nabovati E., Akbari H., Anvari S. (2024). Improving asthma control and quality of life via a smartphone self-management app: A randomized controlled trial. Respir. Med..

[27] Feldman J.M., Kutner H., Matte L., Lupkin M., Steinberg D., Sidora-Arcoleo K., Serebrisky D., Warman K. (2012). Prediction of peak flow values followed by feedback improves perception of lung function and adherence to inhaled corticosteroids in children with asthma. Thorax.

[28] Feldman J.M., Rastogi D., Warman K., Serebrisky D., Arcoleo K. (2025). Peak Flow Feedback Intervention Improves Underperception of Airflow Limitation in Pediatric Asthma: A Randomized Clinical Trial. Ann. Am. Thorac. Soc..

[29] Gupta S., Shabnam, Verma A.K. (2024). Peak expiratory flow rate monitoring can predict asthma exacerbation better as compared to monitoring by symptoms, prospective cohort study. Int. J. Contemp. Pediatr..

[30] Basel Research Centre for Child Health Alex: Design, Development and Evaluation of a Digital Health Assistant for Paediatric Asthma. https://brc.ch/research/alex/.

[31] Bousquet J., Schünemann H.J., Sousa-Pinto B., Zuberbier T., Togias A., Samolinski B., Bedbrook A., Czarlewski W., Hofmann-Apitius M., Litynska J. (2024). Concepts for the Development of Person-Centered, Digitally Enabled, Artificial Intelligence-Assisted ARIA Care Pathways (ARIA 2024). J. Allergy Clin. Immunol. Pr..

[32] Sousa-Pinto B., Costa E.M., Vieira R.J., Klimek L., Czarlewski W., Pfaar O., Bedbrook A., Amaral R., Brussino L., Kvedariene V. (2025). Adherence to Treatment in Allergic Rhinitis During the Pollen Season in Europe: A MASK-air Study. Clin. Exp. Allergy.

